# Chronic Stress Induces Sex-Specific Alterations in Methylation and Expression of Corticotropin-Releasing Factor Gene in the Rat

**DOI:** 10.1371/journal.pone.0028128

**Published:** 2011-11-23

**Authors:** Linda Sterrenburg, Balázs Gaszner, Jeroen Boerrigter, Lennart Santbergen, Mattia Bramini, Evan Elliott, Alon Chen, Bernard W. M. M. Peeters, Eric W. Roubos, Tamás Kozicz

**Affiliations:** 1 Department of Cellular Animal Physiology, Donders Institute for Brain, Cognition and Behaviour, Centre for Neuroscience, Radboud University Nijmegen, Nijmegen, The Netherlands; 2 Department of Anatomy, Pécs University, Pécs, Hungary; 3 Department of Neurobiology, Weizmann Institute of Science, Rehovot, Israel; 4 Faculty of Medicine, Bar Ilan University, Safed, Israel; Baylor College of Medicine, United States of America

## Abstract

**Background:**

Although the higher prevalence of depression in women than in men is well known, the neuronal basis of this sex difference is largely elusive.

**Methods:**

Male and female rats were exposed to chronic variable mild stress (CVMS) after which immediate early gene products, corticotropin-releasing factor (CRF) mRNA and peptide, various epigenetic-associated enzymes and DNA methylation of the *Crf* gene were determined in the hypothalamic paraventricular nucleus (PVN), oval (BSTov) and fusiform (BSTfu) parts of the bed nucleus of the stria terminalis, and central amygdala (CeA).

**Results:**

CVMS induced site-specific changes in *Crf* gene methylation in all brain centers studied in female rats and in the male BST and CeA, whereas the histone acetyltransferase, CREB-binding protein was increased in the female BST and the histone-deacetylase-5 decreased in the male CeA. These changes were accompanied by an increased amount of c-Fos in the PVN, BSTfu and CeA in males, and of FosB in the PVN of both sexes and in the male BSTov and BSTfu. In the PVN, CVMS increased CRF mRNA in males and CRF peptide decreased in females.

**Conclusions:**

The data confirm our hypothesis that chronic stress affects gene expression and CRF transcriptional, translational and secretory activities in the PVN, BSTov, BSTfu and CeA, in a brain center-specific and sex-specific manner. Brain region-specific and sex-specific changes in epigenetic activity and neuronal activation may play, too, an important role in the sex specificity of the stress response and the susceptibility to depression.

## Introduction

Chronic exposure to stressors can result in psychopathologies, of which depression is ranked second in the global burden of disease [1]–[7]. The incidence of depression is sex-specific, as women are affected twice as often as men [8], [9]. The neuronal basis of depression is only fragmentary known but a major neuronal component is corticotropin-releasing factor (CRF) produced by the hypothalamic paraventricular nucleus (PVN), which controls the hypothalamic-pituitary adrenal (HPA-) axis [10], [11]. In depressed people the number of CRF mRNA and CRF peptide-containing PVN neurons is increased [12], [13], which suggests that chronic stressors change the regulatory input system to the PVN. Rodent studies underpin this idea, showing that various forebrain centers control HPA-axis activity, of which the central amygdala (CeA) and the oval subdivision of the bed nucleus of the stria terminalis (BSTov) [14], [15] are of particular interest. Both play a role in the control of mood [16]–[19], host the majority of the brain's CRF neurons [20]–[23] and change their activity upon exposure to chronic stress [24]. Also the fusiform subdivision of the BST (BSTfu) may act in the stress response because it contains CRF, projects to the PVN and BSTov [25] and its lesioning decreases the stress response by the HPA-axis [26]. Recently, we have found that the rat PVN, BSTov, BSTfu and CeA are differentially and sex-specifically affected by acute restraint stress [27]. In the present study we analyzed these brain centers for their possible responses to chronic stress. We hypothesized that a) chronic stress would affect the functioning of the PVN, BSTov, BSTfu and CeA in a sex-specific way to evoke a sex-specific HPA-axis response, b) this response would be borne out at the neuronal gene expression and/or secretory level, and c) these effects would be effectuated, at least partly, by an epigenetic mechanism. Indeed, recent studies indicate the involvement of such a mechanism in stress adaptation. In mice susceptible to chronic social stress, increased CRF mRNA in the PVN is reflected by decreased methylation of the *Crf* gene [28], whereas the other important epigenetic mechanism, histone (de)acetylation, is active in the nucleus accumbens and hippocampus in a mouse model for depression [29], [30].

To test our hypothesis, we exposed rats for 2 weeks to the chronic variable mild stress paradigm (CVMS), a realistic animal model of depression [31]–[36], and subsequently determined various parameters for neuronal activity in the CRF-producing brain centers mentioned above, namely the presence of a) immediate early gene (IEG) transcription factors, c-Fos and FosB by immunocytochemistry, as markers for neuronal activation [37], b) CRF mRNA by *in situ* hybridization, to assess CRF production capacity, c) CRF peptide by immunocytochemistry, indicating CRF storage, d) *Crf* gene methylation by DNA methylation assay, and e) histone-deacetylases (HDAC) 3, 4 and 5, and histone-acetyltransferases (HATs) CREB-binding protein (CBP) and P300/CBP-associated factor (PCAF) by quantitative RT-PCR (Q-RT-PCR) indicating the capacity for epigenetic activity. To reveal sex-specific effects of CVMS exposure, both male and female rats were studied.

## Materials and Methods

### Animals

Seventy two Wistar-R Amsterdam rats (females: 200–250 g, males: 300-350 g), aged 14 weeks, were housed at a light/dark 12/12 h cycle (lights on 07:00 h) at 23°C, with water and food *ad libitum*. They were used for three experiments (n = 24): a) histology (immunocytochemistry and *in situ* hybridization), b) DNA methylation assay, and c) Q-RT-PCR. Body weight was determined at the start of an experiment and at days 5, 10 and 14 of the CVMS protocol. In each experiment 12 rats (6 males, 6 females) were exposed to CVMS for 2 weeks (for protocol, see Information S1A) whereas the other 12 rats (6 males, 6 females) were handled as stressed rats but not exposed to CVMS (controls). Daytime stressors were always administered between 8–9 a.m., and overnight stress exposure started at 6 p.m. and lasted till 6 a.m.. One hour after CVMS exposure on day 14, rats for histology were deeply anesthetized with nembutal (100 mg/kg body weight; Sanofi-Synthélabo, Budapest, Hungary), and under anesthesia a blood sample was taken for corticosterone (CORT) assay. Then, rats were transcardially perfused with 50 ml 0.1 M sodium phosphate-buffered saline (PBS; pH 7.4), for 10 min, followed by 250 ml 4% ice-cold paraformaldehyde in PBS, for 20 min, decapitated, and their brains dissected and postfixed in the paraformaldehyde fixative, for 16 h. For the other two experiments rats were immediately decapitated and their brains frozen till further processing. Since the central stress response in rodents is known to have a circadian nature [38], all decapitations were carried out between 9 and 11 am.

The character of the female stress response depends to some degree on the phase of the estrous cycle [39], [40]. To prevent handling-induced stress, we determined this phase (using vaginal smears) not during the experiment but directly after sacrificing the female rats as described previously [41]–[43]. We have tested successfully our hypothesis that stress affects the functioning of particular stress-sensitive brain centres. Whether this effect is directly on these brain areas or proceeds via a change of the phase of reproductive cycle, cannot be concluded with full certainty. However, such a change is less likely because in the female experimental groups all cycle phases appeared to occur in a rather random fashion (3 pro-estrous, 2 estrous, 1 di-estrous), and statistical analysis (test for normality; [44]) showed that data of all parameters measured did not significantly differ from a normal distribution, and statistical tests (see below) showed that data of all parameters measured were normally distributed and had a low variance not dissimilar from that in males, our conclusions on sex-dependency apply to female rats in general rather than to females in one particular phase of the estrous cycle.

All studies were conducted in accordance with the Directive 86/609/EEC on the protection of Animals used for Experimental and other scientific purposes and the Ethical Codex of Animal Experiments, and were carried out with the approval of the Ethics Committee on Animal Research of Pécs University (approval nr.: BA 02/2000-20-2006).

### CORT assay

For CORT radioimmunoassay, 5 µl blood serum was treated as described previously [45], using ^3^H-corticosterone (12,000 cpm; 90–120 Ci/mmol, NET-399; Perkin-Elmer, Boston, MA) and our CS-RCS-57 CORT antiserum [46]. The inter- and intra-assay co-efficient of variation were 9.2% and 6.4%, respectively, indicating the high reliability of the method.

### Tissue preparation for histology

Fixed brains were transferred to 30% sucrose in PBS, and when completely submerged, frozen on dry ice. Twenty-five µm thick, serial coronal sections between Bregma −0.26 and −3.30 mm [47] were cut on a freezing microtome (Microm, Walldorf, Germany) and kept in sterile antifreeze solution (0.05 M PBS, 30% ethylene glycol, 20% glycerol) at −20°C, till further use. For the PVN and CeA 5 sections were used at the mid-level of each brain nucleus, interspaced by 125 µm and for the BSTov and BSTfu 3 sections were used.

### Immunocytochemistry

Free-floating diaminobenzidine (DAB) immunocytochemistry was carried out as described previously [48] using sera raised in rabbit, to anti-c-Fos (dilution 1∶2000 in PBS; sc-48; Santa Cruz Biotechnology, Santa Cruz, CA), anti-FosB (1∶1000; Santa Cruz) and anti-CRF (1∶2000; kindly provided by Dr W.W. Vale, The Salk Institute, La Jolla, CA). No signals were seen after omission of the first antiserum (immunohistochemistry).

### In situ hybridization

*In situ* hybridization of CRF mRNA was carried out as described previously [49] using *ca.* 40 ng/ml antisense or sense (control; no hybridization signal was seen) cRNA probes transcribed from CRF cDNA (kindly provided by Dr. W.W. Vale), and labeled with DIG-11-UTP (Roche Molecular Biochemicals, Basel, Switzerland). No signals were seen when using sense probes (*in situ* hybridization).

### DNA methylation assay

Genomic DNA was isolated from punches of the PVN, BSTov, BSTfu or CeA, as follows. One mm-thick coronal sections, cut with a razor blade between the cerebellum and both hemispheres using a coronal brain matrix (no. 15007; Ted Pella, Redding, CA), were placed on a chilled mat, and regions of interest punched out with a Harris Unicore Hole 1.0 mm puncher (Ted Pella). Separate punches were made of the PVN, CeA and BST (containing both BSTov and BSTfu). DNA was isolated from the punches using a DNeasy blood & tissue kit and DNeasy mini spin columns (Qiagen, Valencia, CA) and further processed in accordance with the manufacturer's instructions. Bisulfite conversion and pyrosequencing of the promoter region of exon 1 and of the intronic sequence between exons 1 and 2 of the *Crf* gene were performed by EpigenDX, as described previously [50]. The promoter regions contained the CRE site and the AP1, which are important regulatory regions containing CpGs [51]. As other regulatory regions of *Crf* do not have CpGs, these were not analyzed.

### RNA extraction and cDNA synthesis

RNA extraction was carried out as reported before [52]. First strand cDNA was synthesized with 11 ìg RNA dissolved in 11 ìl RNAse-free DEPC containing 5 mU pd(N)6 random primers (Roche), at 70°C for 10 min, followed by double-strand synthesis in 1× strand buffer (Life Technologies, Paisley, UK) with 10 mM DTT, 20 U Rnasin (Promega, Madison, WI), 0.5 mM dNTPs (Roche) and 100 U reverse transcriptase (Superscript II; Life Technologies), for 75 min at 37°C and for 10 min at 95°C.

### Q-RT-PCR

Q-RT-PCR was done as described previously [52] with primers designed using Vector PrimerExpress software (Applied Biosystems, Foster City, CA), based on the respective rat cDNA sequences, according to Information S1B. All data were normalized to 18S mRNA contents.

### Image analysis

For each immunocytochemical reaction as well as for the *in situ* hybridization, image analysis was performed in 5 sections of the PVN and CeA and in 3 sections of the BSTov and BSTfu, at the mid-level of each brain nucleus, interspaced by 125 µm, as described in Information S1C. For the location of these brain areas see Figure S1. In short, numbers of immunoreactive neurons and CRF mRNA containing neurons were counted in all sections and subsequently expressed a mean number per section. A similar procedure was followed for the specific signal density (SSD) of CRF immunostaining and CRF *in situ* signal per neuron, which was determined using Scion Image software (version 3.0b; NIH, Bethesda, MD). In the same way, the SSD of CRF-immunopositive fibers in the BSTfu was measured. The area occupied by CRF immunoreactive fibers in the BSTov was measured in its medial section.

### Statistics

Each parameter is graphically represented as the mean and the standard error of the mean (SEM) of all 6 animals of an experimental group. Means were analyzed with two-way analysis of variance (ANOVA), and if a significant main effect (“stress”, “sex”) or interaction “stress×sex” was found, Fisher's *post hoc* test (Statistica, StatSoft, Tulsa, OK) was carried out. Appropriate transformation of data was applied on the basis of tests for normality [44] and Bartlett's test for the homogeneity of variance [53]. In addition, data on total DNA methylation per brain region were submitted to Wilcoxon's signed rank test [54]. Body weight was analyzed using repeated measures ANOVA with time as within-factor.

## Results

### Physiological parameters

**Figure 1:**
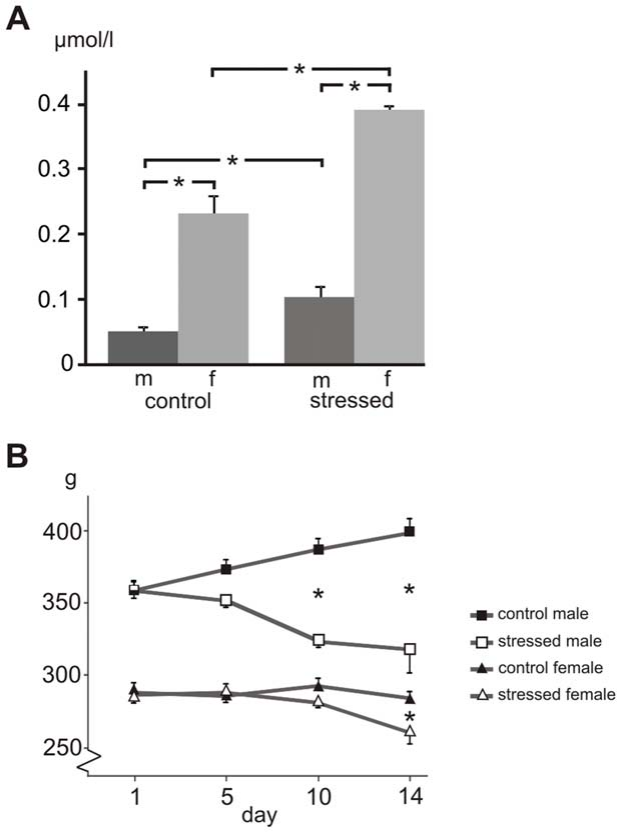
Corticosterone titer and body weight after stress. Corticosterone titer in µmol/l (A) and body weight in grams (B) of control and stressed male (m) and female (f) rats. Means and SEM, n  =  6 per group, * statistical difference between groups indicated, at P < 0.05.

*CORT titer.* Radioimmunoassay of CORT (Fig. 1A) revealed a clear difference between male and female controls (ANOVA: F_1.15_ = 281.4, P<0.00001), the latter having a 2.9x higher CORT titer (P<0.0005). In response to CVMS, both males (2.0x; P<0.05) and females (1.7x, P<0.0005) showed a higher CORT titer, underpinning the stress effect (F_1.15_ = 56.0, P<0.00001).

*Body weight.* Also body weight gain demonstrated clear effects of stress (F_1.19_ = 58.0, P<0.00001) and sex (F_1.19_ = 501.7, P<0.00001; Fig. 1B). While control males showed a body weight gain of about 12% (P<0.005), stressed males lost about 6% of their body weight at day 14 vs. day 0 (P<0.005). Females differed from males in that they had a 20% lower body weight at the start of the experiment (P<0.0001). Female controls did not gain weight during the experiment, but stressed females at day 14 showed a 10% decrease in body weight compared to day 0 (P<0.05).

### Gene expressions and secretory activity

**Figure 2:**
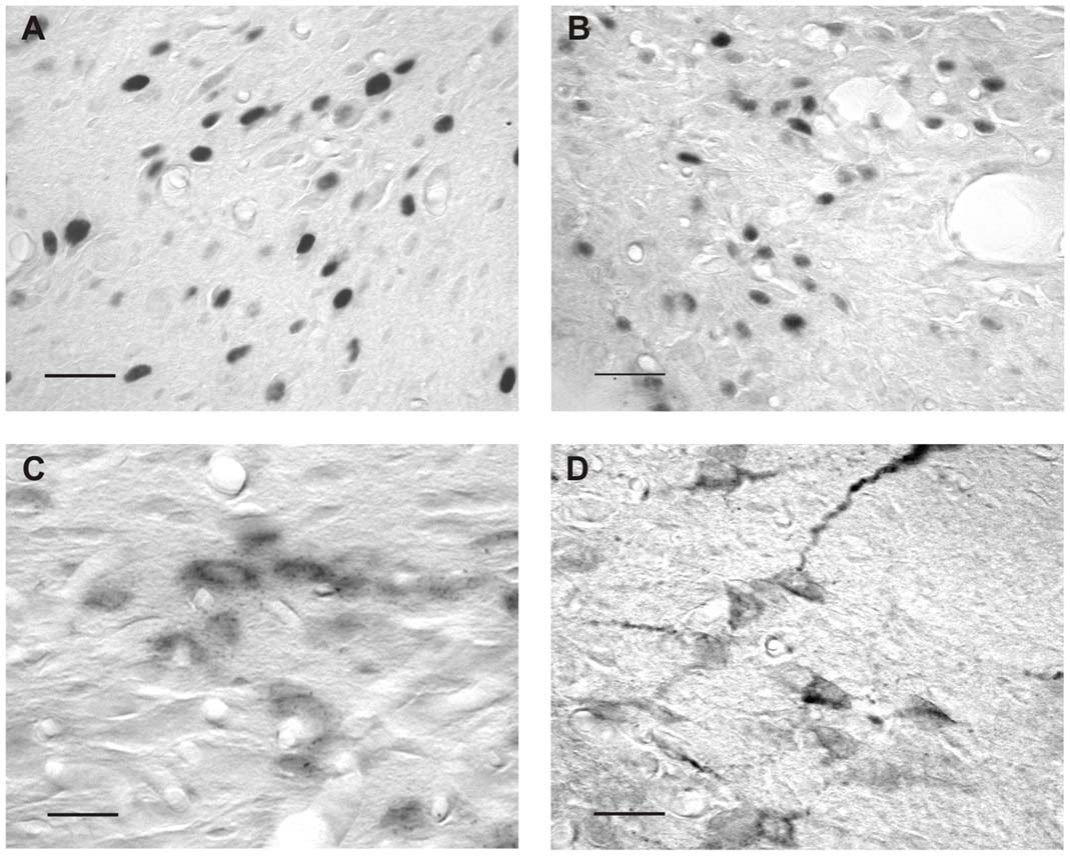
Detailed images of immunocytochemistry and *in situ* hybridization. Images representative for immunocytochemistry of c-Fos (A), FosB (B), for *in situ* hybridization of CRF mRNA (C) and immunocytochemistry of CRF (D) in the rat brain, taken in the hypothalamic paraventricular nucleus. Scale bars  =  20 µm.

Immunocytochemistry and *in situ* hybridization were performed for all four brain regions. Detailed images of this can be found in Figure 2.

**Figure 3:**
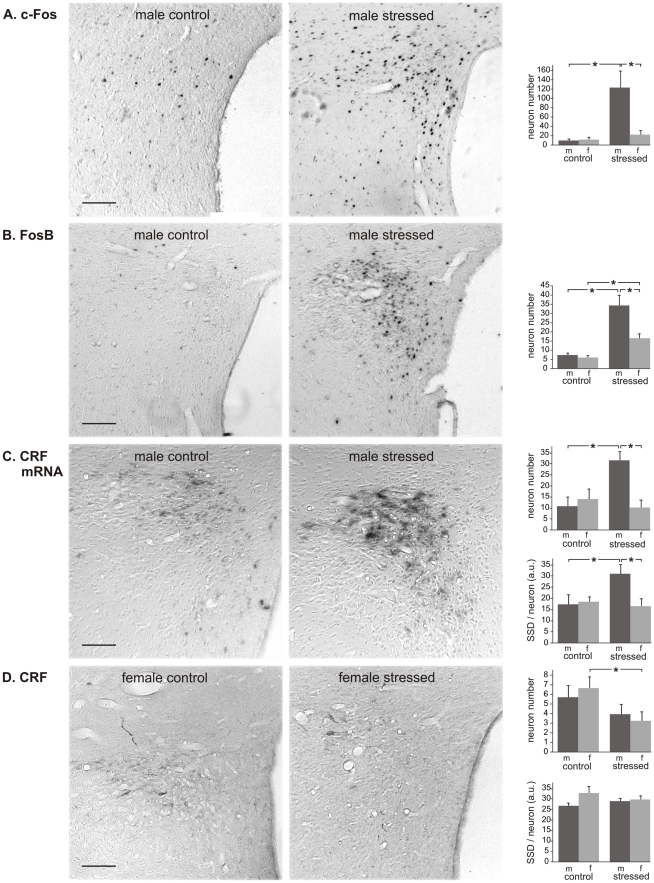
Paraventricular nucleus of the hypothalamus. Paraventricular nucleus of the hypothalamus in control and stressed male (m) and female (f) rats, with representative images, and numbers per section of neurons stained for c-Fos (A), FosB (B), CRF mRNA (C) and CRF (D), and specific staining density per neuron (SSD) in arbitrary units (a.u.; C and D). Means + SEM, n  =  6 per group, * P < 0.05. Scale bars  =  50 µm.

*PVN.* To assess the influence of CVMS on the expression of IEGs, we used c-Fos- and FosB-immunocytochemistry. For c-Fos (Fig. 3A) the ANOVA showed effects of stress (F_1.17_ = 17.6; P<0.01) and sex (F_1.17_ = 7.3; P<0.05), and a sex×stress interaction (F_1.17_ = 9.0; P<0.01). *Post hoc* analysis revealed that males had a dramatically higher number of c-Fos-positive cells after CVMS (13.2x; P<0.0005), whereas females did not react to CVMS (P > 0.05). Also for FosB effects of stress (F_1.19_ = 46.7; P<0.00001), sex (F_1.19_ = 8.8; P<0.001) and a sex×stress interaction (F_1.19_ = 5.1; P<0.05) were present, but here both males and females showed increased numbers of FosB-positive cells upon CVMS (Fig. 3B). Males mounted a substantially stronger response than females (males: 4.7x, females: 2.7x; P<0.0005). To determine if CVMS had affected the production and storage of CRF, *in situ* hybridization of CRF mRNA and immunocytochemistry of CRF peptide were carried out, respectively (Fig. 3C, 3D). Males showed a higher number of CRF mRNA-positive cells after CVMS (2.9x; P<0.005; Fig. 3C), but in females no significant reaction to CVMS was found (P>0.05). Similarly, only males revealed a stress-induced increase in the amount of mRNA in individual neurons (SSD: 1.8x, P<0.05), whereas females did not show a stress effect. As to CRF immunocytochemistry, after CVMS the number of CRF-immunoreactive neurons decreased by 2.0x in females (P<0.05; Fig. 3D), whereas no stress effect could be detected in males (P = 0.32). The SSD of CRF-stained neurons did not significantly differ between the experimental groups.

**Figure 4:**
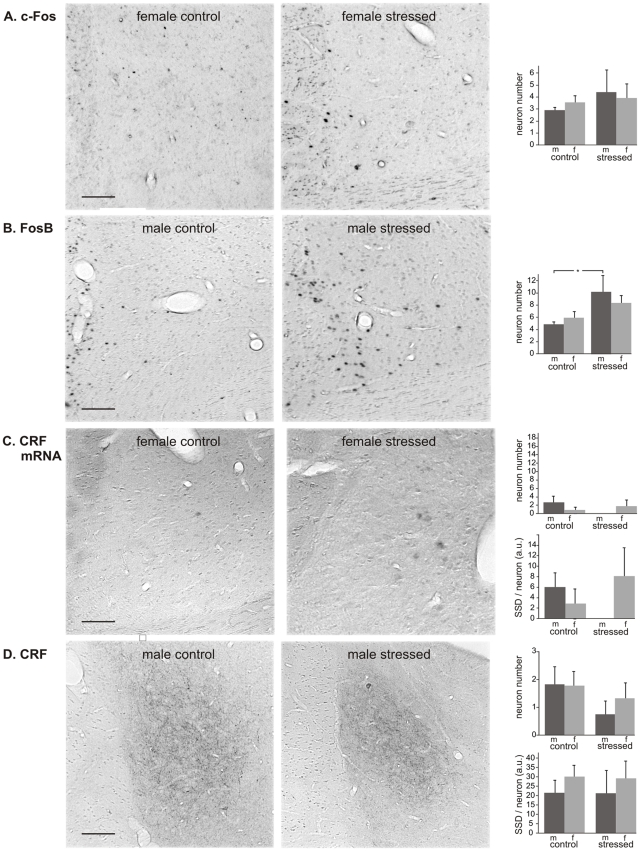
Oval bed nucleus of the stria terminalis. Oval bed nucleus of the stria terminalis (BSTov) in control and stressed male (m) and female (f) rats, with representative images, and numbers per section of neurons stained for c-Fos (A), FosB (B), CRF mRNA (C) and CRF (D), and specific staining density per neuron (SSD) in arbitrary units (a.u.; C and D). Means + SEM, n  =  6 per group, * P < 0.05. Scale bars  =  50 µm.

*BSTov*. With respect to c-Fos, no effect of CVMS was found in either sex (Fig. 4A). In contrast, for the number of FosB-positive cells ANOVA showed a clear stress effect (F_1.18_ = 7.5; P<0.05). *Post hoc* analysis revealed a 2.1x higher number of c-Fos positive neurons in males exposed to CVMS vs. controls (Fig. 4B; P<0.05). No CVMS effect was detected in females. Furthermore, also no stress effect was seen in either sex as to the number and SSD of neurons positive for CRF mRNA (Fig. 4C) or for CRF peptide (Fig. 4D). The size of the human BSTov reveals a sexual dimorphism, being larger in males than in females [55]. Therefore, we tested if such dimorphism would hold for the CRF-immunoreactive BSTov in our rats. ANOVA did not reveal an effect of sex (F_1.16_ = 0.46, p>0.05) or of stress (F_1.16_ = 0.22, p>0.05) on the surface area of this nucleus.

**Figure 5:**
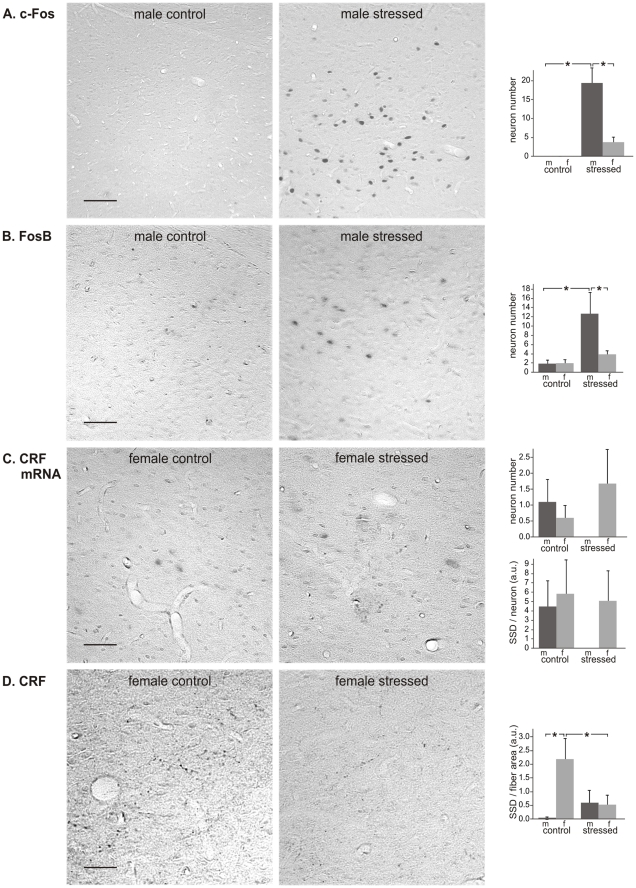
Fusiform bed nucleus of the stria terminalis. Fusiform bed nucleus of the stria terminalis (BSTfu) in control and stressed male (m) and female (f) rats, with representative images, and numbers per section of neurons stained for c-Fos (A), FosB (B), CRF mRNA (C) and CRF (D), and specific staining density per neuron (SSD) in arbitrary units (a.u.; C and D). Means + SEM, n  =  6 per group, * P < 0.05. Scale bars  =  50 µm.

*BSTfu.* We observed a clear CVMS-induced increase in the number of c-Fos-positive neurons in males (P<0.00001; Fig. 5A), whereas in females no effect of CVMS was detectable. A similar sex-specific situation was encountered for the number of FosB-immunopositive neurons (Fig. 5B), as these had only been recruited in males (6.6x; P<0.005). Similarly to the BSTov, neither the number nor the SSD of CRF mRNA-positive neurons significantly differed between control and CVMS groups (Fig. 5C). CRF-positive neurons were not seen, but a densely CRF-staining fiber network was present throughout the BSTfu (Fig. 5D). The SSD of these fibers revealed a strong sex difference among control animals, female controls showing much stronger staining than male controls (P<0.05). CVMS has also resulted in a strong reduction in the SSD of CRF-immunoreactive fibers in females (4.1x; P<0.05), whereas in males such an effect was not significant.

**Figure 6:**
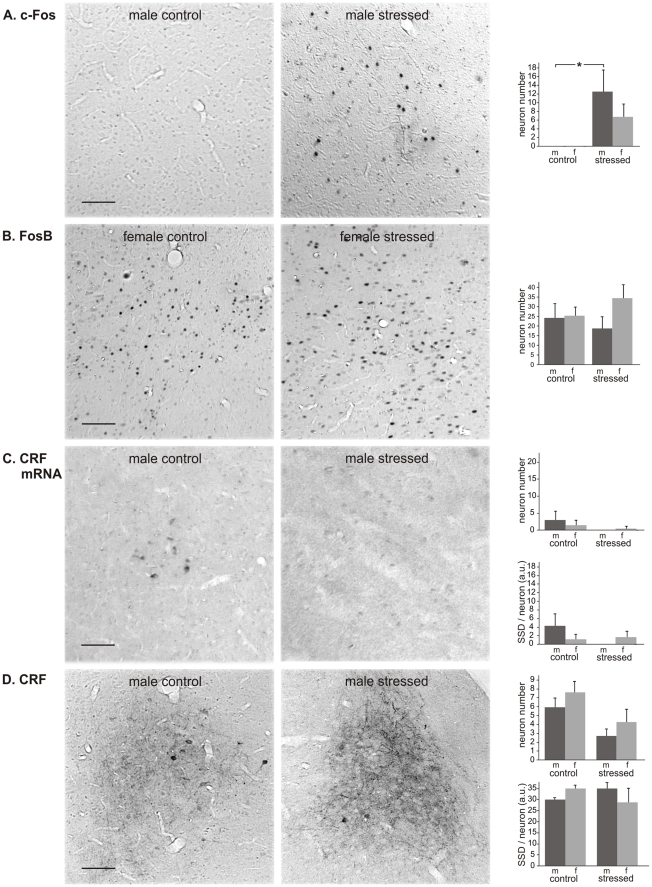
Central amygdale. Central amygdala (CeA) in control and stressed male (m) and female (f) rats, with representative images, and numbers per section of neurons stained for c-Fos (A), FosB (B), CRF mRNA (C) and CRF (D), and specific staining density per neuron (SSD) in arbitrary units (a.u.; C and D). Means + SEM, n  =  6 per group, * P < 0.05. Scale bars  =  50 µm.

*CeA.* The number of c-Fos-stained neurons was higher in CVMS-exposed males than in controls (P<0.05; Fig. 6A), whereas no CVMS effect could be shown in females. The number of FosB-positive neurons did not differ among groups (Fig. 6B). The number of CRF mRNA- and CRF-positive neurons and their SSD were similar for males and females, but in both sexes there was a strong tendency that the number of CRF-immunoreactive neurons had decreased upon stress (−54% in males; P = 0.10, and −43% in females; P = 0.064; Fig. 6C, 6D).

### Epigenetic factors

**Figure 7:**
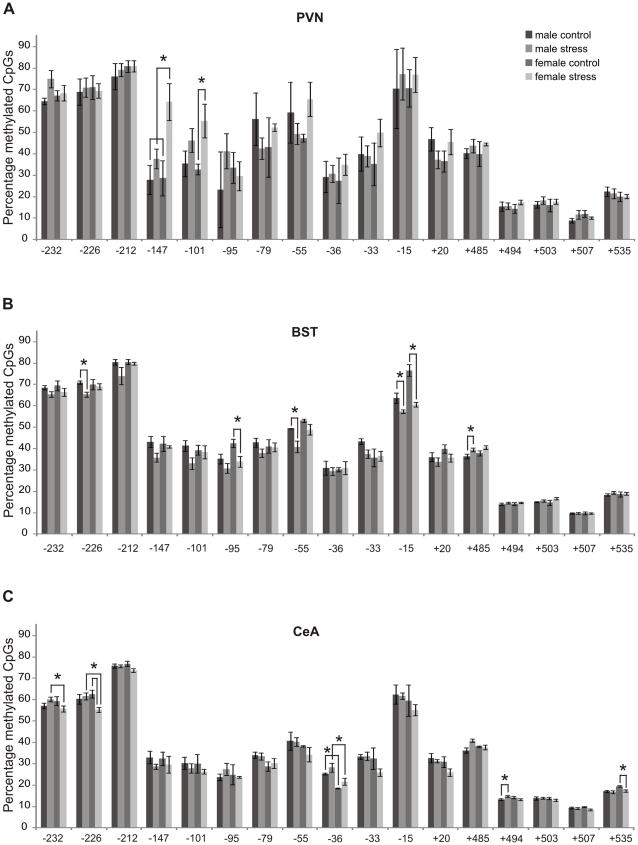
Methylation of the *Crf* gene. Methylation of the *Crf* gene in the PVN (A), BST (B) and CeA (C) in control and stressed male and female rats. CpGs, cytosine-phosphate-guanine sites. Means±SEM, n  =  6 per group, * P < 0.05.

*PVN.* The total DNA methylation of the *Crf* gene over all cytosine-phosphate-guanine sites (CpGs) was consistently higher in stressed females than in female controls (P<0.01), whereas no stress effect was demonstrable in males. When individual CpGs were considered (Fig. 7A), CVMS appeared to have increased DNA methylation in CpG-147 and CpG-101. In both CpGs the effect was sex-specific, as not males but females showed a significant increase (CpG-147: 2.2x; P<0.05, and CpG -101: 1.7x; P<0.05). For the other CpGs no effect of CVMS was seen. As to histone acetylation and deacetylation, Q-RT-PCR analysis did not reveal a stress effect on the mRNA amounts of HDAC 3, 4 and 5, PCAF and CBP (Table 1).

**Table 1:** Histone acetylation.

Brain centre	Enzyme	Male control	Male stressed	Female control	Female stressed	
PVN	HDAC 3	1.6±0.2	1.6±0.1	2.0±0.3	1.9±0.2	
	HDAC 4	2.0±0.3	2.4±0.4	2.0±0.2		2.6±0.4
	HDAC 5	1.4±0.2	1.9±0.3	1.9±0.2		1.7±0.1
	CBP	0.8±0.1	0.8±0.1	0.8±0.1		1.4±0.3
	PCAF	0.7±0.2	0.6±0.1	0.5±0.0		0.7±0.1
BST	HDAC 3	1.6±0.1	1.4±0.2	1.7±0.3		1.9±0.3
	HDAC 4	3.2±0.9	2.5±0.5	3.4±0.8		4.0±0.6
	HDAC 5	1.3±0.1	1.4±0.2	1.5±0.1		1.4±0.1
	CBP	0.6±0.1	0.8±0.2	0.6±0.1		1.0±0.1*
	PCAF	0.5±0.1	0.6±0.1	0.5±0.1		0.5±0.1
CeA	HDAC 3	1.6±0.2	1.5±0.2	1.8±0.2		1.6±0.2
	HDAC 4	1.7±0.1	2.1±0.3	2.0±0.2		2.5±0.2
	HDAC 5	1.8±0.2	1.1±0.1*	1.4±0.2		1.2±0.1
	CBP	0.8±0.1	0.9±0.3	0.5±0.1		0.6±0.1
	PCAF	0.6±0.1	0.7±0.1	0.6±0.1		0.8±0.1

Q-RT-PCR for HDAC 3, 4 and 5, PCAF and CBP mRNAs in the PVN, BST and CeA of control and stressed male and female rats. Means±SEM, n  =  6.

*P<0.05 compared with control.

*BST.* The data on epigenetic markers apply to the whole BST. Overall, *Crf* methylation was less strong in stressed males than in male controls (P<0.005), whereas females did not show a stress effect. However, when looking at individual CpGs, 5 CpGs had changed in response to CVMS (Fig. 7B). At CpG-15 DNA methylation was lower in both males (∼10%; P<0.05) and females (∼30%; P<0.05). A sex-specific effect was found for 4 CpGs. At CpGs −226 and −55, stress resulted in ∼10–20% lower DNA methylation in males (P<0.05), whereas stress did not result in changes in these CpGs in females. On the other hand, at CpG-95 females showed ∼30% lower methylation after stress (P<0.05), whereas in males no change was observed. At CpG +485, stressed males demonstrated ∼10% higher DNA methylation than controls (P<0.05), whereas a stress effect in females was lacking. Q-RT-PCR revealed an increased amount of CBP mRNA (1.9x; P<0.05) upon CVMS in females, whereas no stress effect was seen in males (Table 1). For the other enzymes studied no stress effects were found.

*CeA.* When the total methylation of the *Crf* gene in the CeA was considered, stressed females showed less methylation than stressed males (P<0.001). In females total methylation was reduced by stress (P<0.005) whereas methylation in males was not. However, when individual CpGs were considered, the picture appeared to be more complex. CVMS had induced changes in 5 CpGs (Fig. 7C). In both CpG -232 and CpG -226 stressed males revealed stronger methylation than stressed females. In addition, in CpG -226, stressed females had ∼10% lower methylation than control females (P<0.05). At CpG-36 males showed stronger methylation than females, both in the control situation and after CVMS. At CpG +494, CVMS led to ∼10% more methylation in males (P<0.05), whereas females did not react to the stressor. At CpG +535, finally, stress had decreased methylation in females only, by ∼10% (P<0.05). Q-RT-PCR showed that CVMS had decreased the amount of HDAC 5 mRNA in males (by 1.6x; P<0.05), whereas females did not show a stress effect on histone acetylation and deacetylation epigenetic markers (Table 1).

## Discussion

We show that exposing rats to CVMS activates the HPA-axis, as in both male and female stressed rats the CORT titer was increased and body weight gain was decreased. These results indicate that the CVMS paradigm resulted in the chronic activation of the HPA-axis, a phenomenon often seen in depression [1], [10], [12]. While there are several reports on chronic stress-induced *Crf* expression and increased CRF peptide release in the PVN, such information on extrahypothalamic brain centers is scarce [56]–[59]. Moreover, nothing is known about the possible sex-specificity of these phenomena and their possible dependence on epigenetic mechanisms. Here, we provide new evidence that chronic stress, as applied by the CVMS paradigm a) affects the functioning of the PVN, BSTov, BSTfu and CeA to evoke a sex-specific HPA-axis response, b) alters neuronal gene and peptide expressions, and c) changes epigenetic-associated factors. Moreover, we show that many of these stress effects are brain center- and sex-specific. Below we will discuss these conclusions in more detail.

### Technical considerations

*The CVMS paradigm.* Previously we have performed a study exposing rats to acute restraint stress and analyzed the same parameters and brain centers to assess possible sex-specific acute stress-induced responses of CRF neurons in the rat forebrain [27]. As an added value for the present study, we also aimed to assess the effect of previous stress history on the animal's response to an acute psychological stressor (restraint) and to compare the animal's response to acute restraint- *vs.* chronic variable mild stress-related responses of forebrain CRF expressing neurons (see later in discussion). Therefore, we have modified the CVMS paradigm so that the rats were exposed to restraint stress on the 14^th^ day, and were sacrificed 2 hours post-stress. This approach has the advantage that the last stressor in both cases was a 60 min restraint, thus allowing direct comparison of the animal's response to restraint stress with or without a chronic stress history. One could however argue that because rats were sacrificed 2 hours after the last stress exposure in our CVMS paradigm, the effects found could have been (particularly) caused by the last stressor of the series of unpredicted mild stressors, *i.e.,* 60 min of restraint stress. However, our data do not support this, because in the PVN, acute restraint stress induces c-Fos in both sexes and FosB only in females [27], whereas in the present study CVMS increases c-Fos in males only and FosB in both sexes. In addition, it is well known that the effects of restraint stress alone are different from that of CVMS exposure [27], [60]. Therefore, we argue that the effects found in our study are due to chronic stress exposure and not to acute restraint stress.

*The use of c-Fos and FosB.* Immunoreactivities to the IEGs c-Fos and FosB are well-established markers for neuronal activation [61], [62]. c-Fos is induced shortly after exposure to a wide range of stressors [63] including chronic stressors that increase its expression in, for example, the PVN [64], [65]. As to FosB, our anti-FosB serum recognizes various of its splice variants [66], including full-length FosB, which is induced by acute stress, and deltaFosB, which is increased after chronic stress [66] and is thought to play a role in long-term adaptation [61].

### PVN

The present demonstration of CVMS-induced increase in the number of PVN neurons expressing FosB in both sexes and c-Fos in males underlines the ability of the CVMS paradigm to induce (several sets of) genes in the PVN [57], [61]. Most likely, genes induced include *Crf*, because we here show, in line with previous studies [33], [34], in males, a CVMS-induced increase in the PVN content of CRF mRNA. Since this increase is not concomitant with an increased storage of CRF peptide, it seems that CVMS activates CRF production in the PVN neuronal cell body with a similar strength as CRF export from the cell body toward the axons, in this way leaving the net amount of CRF stored in the cell body unchanged. Experimental proof that such equal stimulation of CRF production and CRF export would be concomitant with an, expected, (equal) increase in CRF release, awaits further proof.

CVMS-induced increased production of CRF seems to be specific for male rats, because CVMS does not influence CRF mRNA contents in the female PVN, a sex difference previously observed by Duncko et al. [35]. However, unlike males, females did reveal a CVMS effect on the amount of stored CRF; as this amount decreased, but the amount of CRF mRNA did not change, it would seem that the PVN increases CRF release. Obviously, this would rapidly lead to CRF exhaustion and, hence, to reduced CRF release by females. It would seem interesting to test whether such a sex difference in the ability to maintain CRF release upon chronic stress exposure underlies the sex difference in the stress response by the HPA-axis and, for that matter, the sex difference in human depressive behavior [8], [9], [67]–[69]. Nonetheless, the chronic activation of the PVN in response to unpredictable CVMS is in clear contrast to the habituating response of this nucleus to homotypic chronic (restraint) stress [70], which suggests that the character of the PVN stress response depends for a substantial part on the nature of the chronic stressor. In response to acute restraint stress, similarly to CVMS an increased amount of CRF mRNA was observed in males, but the amount of CRF peptide did not change in either sex [56].

**Figure 8:**
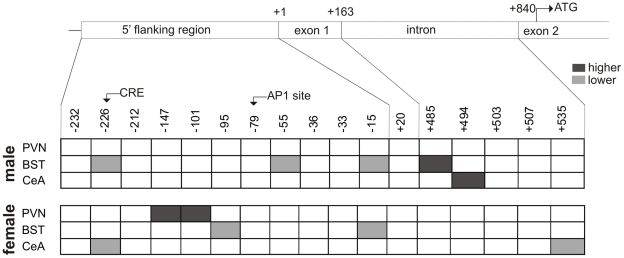
Summary of methylation data. Panel summarizing methylation of the *Crf* gene at sites in 5′ flanking region, exon 1, intron and exon 2 of *Crf,* in the PVN, BST and CeA. Dark box: methylation increase compared with respective control. Light gray box: methylation decrease compared with respective control.

With respect to the possible involvement of epigenetic markers in the response of the PVN to CVMS, in the male PVN no effect of CVMS on histone acetylation and DNA methylation was seen. This may indicate that the presently demonstrated increase in CRF mRNA expression in this nucleus upon CVMS does not depend on an epigenetic mechanism. Still, such involvement cannot be fully ruled out as in our punches other non-CRF containing neurons may have “diluted” the histone acetylation and DNA methylation signals below detection level. Meanwhile, the CVMS-induced increase in females of *Crf* methylation at CpGs −101 and −147 (Fig. 8) may well indicate epigenetic repression of *Crf* transcription and, therefore, explain the absence of an increase in CRF mRNA expression upon CVMS. Since corticosterone is high in females, this might have resulted in an increased negative feedback on the PVN, possibly via increased DNA methylation, accounting for the absence of an increase in CRF mRNA after stress.

### Limbic centers

We show that CVMS activates neurons in the BSTov, BSTfu and CeA in the male rat. This finding indicates that an involvement of these brain centers in the chronic stress response is restricted to males, which is in line with the above discussed sex difference in the way the PVN reacts to CVMS. Extrapolating our results to the human situation, absence of a response in the BSTov, BSTfu and CeA in females could account for the higher incidence of depression in women than in men [8], [9]. However, it should be noted that the presently found changes in reactivity to CVMS by the male BSTov, BSTfu and CeA are largely restricted to the induction of c-Fos and FosB, whereas no changes were observed in the neuronal contents of CRF mRNA and CRF peptide, main factors in the HPA-axis stress response. This indicates that the BSTov, BSTfu and CeA are indeed involved in the animal's response to CVMS, but that possibly neurons containing other neurotransmitters were activated. In support of this latter, it is well established that acute stress specifically recruits GABA/Met-enkephalin neurons in the BSTov and CeA [71], [72], and glutamate/Met-enkephalin neurons in the BSTfu [73], [74]. However, the role and involvement of these limbic, non-CRF neuronal populations in the animals stress response remains elusive, and should be addressed in future studies.

Like in the case of the PVN, these results may be specific for CVMS, because Kim et al. [56] showed that chronic stressors stronger than CVMS do increase CRF mRNA in the male BSTov. On the other hand, the same authors found that chronic stress failed to affect CRF mRNA contents of the male BSTfu [57], suggesting that the neuronal sensitivity to stressors is not only sex- and stressor- but also brain area-dependent.

The amount of CRF in the fiber network of the BSTfu decreased in females in response to CVMS. However, whereas the BSTfu has numerous connections with other brain regions including the PVN [25], it remains to be determined whether the CRF-immunoreactive fibers are BSTfu afferents or efferents towards the BSTfu and, therefore, if the CVMS-induced decrease in their immunoreactivity relates to a change in either input or output from this nucleus. In humans, males have a larger BSTov than females [55]. Apparently, in our rats such a sexual dimorphism does not exist for the CRF-containing part of the BST (this study) nor for its vasoactive intestinal polypeptide- and PACAP-containing neurons [75].

A chronic stress-induced decrease in HDAC5 concomitant with a hypersensitive stress response occurs in the nucleus accumbens [29]. In the present study, we reveal decreased HDAC5 mRNA in the male CeA upon CVMS, whereas in the female BST the stressor increases mRNA of another epigenetic marker, histone acetyltransferase CBP. Therefore, in both forebrain centers CVMS might stimulate an epigenetic process that enables long-term gene expression. Since the BST and CeA do not react to CVMS with a change in CRF mRNA, epigenetic activity would seem to concern other genes than *Crf*. More specifically, the CVMS-induced decrease in HDAC5 mRNA in the male CeA might account for the observed increase in c-Fos content.

As to DNA methylation in the forebrain, we have found that CVMS results in decreased *Crf* methylation in the male BST, at three CpGs in the promoter region, and in increased methylation at an intronic CpG between exons 1 and 2. Also the female BST revealed CVMS effects on *Crf* CpGs methylation sites, but these were exclusively inhibitory and concerned only two CpGs, which differed from the affected CpGs in the PVN (Fig. 7; see also above). These differences in CVMS-induced methylation among sexes and brain centers indicate that CVMS affects *Crf* methylation in a sex- and brain center-specific way as to type (inhibitory *vs*. stimulatory) and CpG site. In view of the absence of CVMS-induced changes in CRF mRNA and peptide contents, elucidation of the functional significance of these epigenetic differences in terms of *Crf* transcription in the forebrain nuclei is of strong interest.

### Conclusions

We have supported our hypothesis that chronic stress, induced by the CVMS paradigm, recruits CRF-producing neurons in the PVN, BSTov, BSTfu and CeA, in a brain center- and sex-specific manner that is clearly different from the responses of these brain centers induced by acute restraint stress [27]. In addition, CVMS also leaves a brain center- and sex-specific epigenetic footprint that may account for the observed differential responses by these neurons to CVMS. The CVMS-induced increase in *Crf* methylation in PVN and the absence of a response of the BSTov, BSTfu and CeA in females could play a role in the mechanism behind the higher incidence of depression in women. Determining the nature of this mechanism is beyond the scope of the present study, but it may be relevant to note that chronic mild stress is known to affect circadian rhythmicity of locomotion in rats [36]. Therefore, it would be interesting to study if the presently found sex- and brain center-specific effects of CVMS on neuronal activities, reflect, at least for a part, disturbed circadian activity of these centers, as such disturbances of brain activity have been presumed to mediate stressor-induced brain disorders like depression [38], [76].

In conclusion, our study contributes to the emerging insight that epigenetic mechanisms could play a role in mounting the central response to chronic stress and, more specifically, indicates that chronic stress can modulate the programming of CRF-producing forebrain centers involved in the long-term HPA-axis stress response. We propose that the mammalian brain possesses sex-specific and epigenetically controlled mechanisms to promote successful stress adaptation and that, consequently, deregulation of these mechanisms in one or more of their constituting stress-sensitive brain centers contributes to the etiology of stress-associated and sex-specific mood disorders, such as depression.

## Supporting Information

Figure S1 **Schematic representation of the sampling sites in the rat brain.** Schematic representation of the sampling sites in the rat brain of the paraventricular nucleus of the hypothalamus (PVN), oval (BSTov) and fusiform (BSTfu) subdivisions of the bed nucleus of the stria terminals, and central amygdala (CeA). Modified after [47].
(TIF)

Information S1 **Chronic variable mild stress paradigm used, primer sequences used for quantitative RT-PCR, and image analysis.**
(DOC)
