# Supplementary figures and images for: Chronic Stress Induces Sex-Specific Alterations in Methylation and Expression of Corticotropin-Releasing Factor Gene in the Rat
Source: PLoS One. 2011 Nov 23;6(11):e28128. doi: 10.1371/journal.pone.0028128 (PMC3223222; doi:10.1371/journal.pone.0028128)

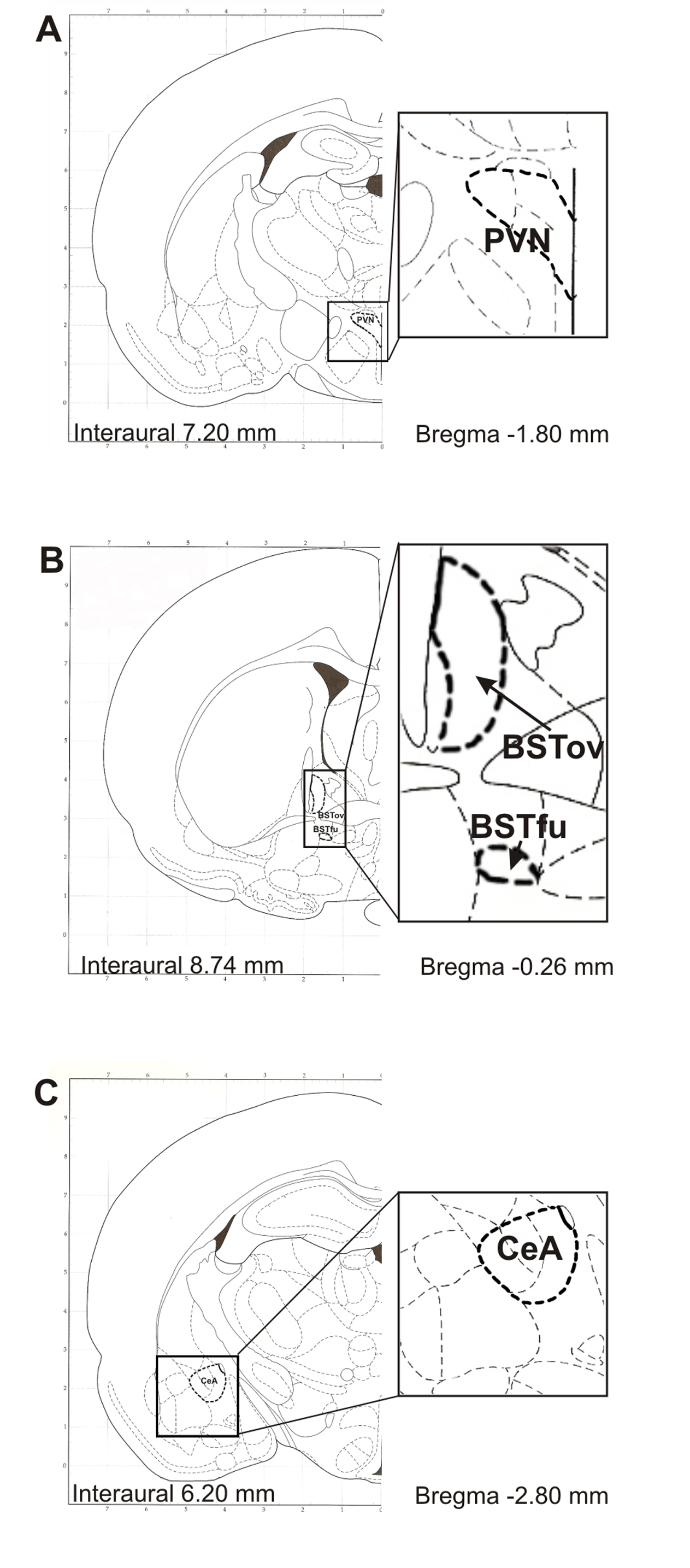

Supplement: Figure S1 — Schematic representation of the sampling sites in the rat brain. Schematic representation of the sampling sites in the rat brain of the paraventricular nucleus of the hypothalamus (PVN), oval (BSTov) and fusiform (BSTfu) subdivisions of the bed nucleus of the stria terminals, and central amygdala (CeA). Modified after [47]. (TIF) [file pone.0028128.s001.tif]
