# Supplementary material for: Chronic Stress Induces Sex-Specific Alterations in Methylation and Expression of Corticotropin-Releasing Factor Gene in the Rat
Source: PLoS One. 2011 Nov 23;6(11):e28128. doi: 10.1371/journal.pone.0028128 (PMC3223222; doi:10.1371/journal.pone.0028128)
Supplement: Information S1 — Chronic variable mild stress paradigm used, primer sequences used for quantitative RT-PCR, and image analysis. (DOC) [file pone.0028128.s002.doc]

**Information S1**

| **Day** | **Stress type** |
| --- | --- |
| 1 | Swim stress, 2 min (4 °C); humid sawdust, 3 h |
| 2 | Food/water deprivation, permanent |
| 3 | Lights on, overnight; humid sawdust, permanent |
| 4 | Lights off, 180 min; swim stress, 2 min (4 °C) |
| 5 | Food/water deprivation, overnight; isolation, overnight |
| 6 | Cold isolation (4 °C), 15 min; lights off, 120 min |
| 7 | Swim stress, 4 min (12 °C); food/water deprivation, overnight |
| 8 | Inverted light/dark cycle; humid sawdust, overnight |
| 9 | Constant light, overnight; food/water deprivation, overnight |
| 10 | Lights off, 180 min; humid sawdust, permanent |
| 11 | Isolation, overnight; food/water deprivation, overnight |
| 12 | Restraint stress, 60 min; lights on, overnight |
| 13 | Inverted light/dark cycle; food/water deprivation, overnight |
| 14 | Humid sawdust, 3 h; restraint stress, 60 min |

A. Chronic variable mild stress paradigm used.

B. Primer sequences used for quantitative RT-PCR. F, forward; R, reverse; HDAC, histone deacetylase; CBP, CREB-binding protein; PCAF, P300/CBP-associated factor.

| **Primer name** | **5'** → **3'** |
| --- | --- |
| HDAC 3 F | GCCAAGACCGTGGCGTATT |
| HDAC 3 R | GTCCAGCTCCATAGTGGAAGT |
| HDAC 4 F | CAATCCCACAGTCTCCGTGT |
| HDAC 4 R | CAGCACCCCACTAAGGTTCA |
| HDAC 5 F | TGTCACCGCCAGATGTTTTG |
| HDAC 5 R | TGAGCAGAGCCGAGACACAG |
| CBP F | GGACCTGGGATCTGCATGAA |
| CBP R | TCCAGCAGCCCCAAGAGA |
| PCAF F | CACGCTCAAGAACATCCTGCA |
| PCAF R | TCGCTGTAAGTCCGCCATGAATA |
| 18S F | GTAACCCGTTGAACCCCATT |
| 18S R | CCATCAATCGGTAGTAGCG |

**C.** *Image analysis*

CRF mRNA expression and immunoreactivity of CRF, c-Fos and FosB were assessed in 5 sections of the PVN and the CeA and in 3 sections of the BSTov and BSTfu, at the mid-level of each brain nucleus, interspaced by 125 µm. Digital images were taken with the Leica DMRBE microscope (at 1,200 x 1,600 dpi). Numbers of immunoreactive neurons were counted per section and then averaged over all sections. The specific signal density (SSD) of CRF-staining per neuron, was determined using Scion Image software (version 3.0b; NIH, Bethesda, MD, USA), averaged over 10 randomly taken neurons, corrected for background staining in the same section outside the brain area, and finally averaged over all sections. In the same way, the SSD of CRF-immunopositive fibers in the BSTov, BSTfu and CeA was measured.
